# Aluminium in Brain Tissue in Multiple Sclerosis

**DOI:** 10.3390/ijerph15081777

**Published:** 2018-08-18

**Authors:** Matthew Mold, Agata Chmielecka, Maria Raquel Ramirez Rodriguez, Femia Thom, Caroline Linhart, Andrew King, Christopher Exley

**Affiliations:** 1 The Birchall Centre, Lennard-Jones Laboratories, Keele University, Staffordshire ST5 5BG, UK; m.j.mold@keele.ac.uk (M.M.); raquel.ramirez3@hotmail.com (M.R.R.R.); 2 Life Sciences, The Huxley Building, Keele University, Staffordshire ST5 5BG, UK; aggychmi@gmail.com (A.C.); femiathom@hotmail.com (F.T.); 3 Department of Medical Statistics, Informatics and Health Economics, Medical University of Innsbruck, A-6020 Innsbruck, Austria; Linhart.Caroline@i-med.ac.at; 4 Department of Clinical Neuropathology, Kings College Hospital, London SE5 9RS, UK; andrewking@nhs.net

**Keywords:** multiple sclerosis, human exposure to aluminium, human brain tissue, TH GFAAS, aluminium-specific fluorescence

## Abstract

Multiple sclerosis (MS) is a devastating and debilitating neurodegenerative disease of unknown cause. A consensus suggests the involvement of both genetic and environmental factors of which the latter may involve human exposure to aluminium. There are no data on the content and distribution of aluminium in human brain tissue in MS. The aluminium content of brain tissue from 14 donors with a diagnosis of MS was determined by transversely heated graphite furnace atomic absorption spectrometry. The location of aluminium in the brain tissue of two donors was investigated by aluminium-specific fluorescence microscopy. The aluminium content of brain tissue in MS was universally high with many tissues bearing concentrations in excess of 10 μg/g dry wt. (10 ppm) and some exceeding 50 ppm. There were no statistically significant relationships between brain lobes, donor age or donor gender. Aluminium-specific fluorescence successfully identified aluminium in brain tissue in both intracellular and extracellular locations. The association of aluminium with corpora amylacea suggests a role for aluminium in neurodegeneration in MS.

## 1. Introduction

Multiple sclerosis (MS) is a chronic, immune-mediated, demyelinating disease of the central nervous system of unknown aetiology. Despite some progress, advances in understanding the pathogenesis of MS remain frustratingly slow [1]. Effective treatments for MS are rare [2,3]. The consensus remains that MS is likely to involve both genetic and environmental factors acting either in isolation or together in various disease phenotypes. Human exposure to aluminium [4] is identified as a possible contributor to MS. Individuals with relapsing remitting (RRMS) and secondary progressive (SPMS) MS were shown to excrete large amounts of aluminium in their urine [5], an observation recently built upon and confirmed in individuals with SPMS [6]. The origin in the body of excreted aluminium, identified in aforementioned research, is unknown; although it may be brain tissue as myelin [7] and oligodendrocytes [8] are specific targets in animal models of aluminium intoxication. However, while data demonstrate the presence of aluminium in human brain tissue in neurodegenerative/neurodevelopmental disease [9,10], there are no such data for MS. Herein we have carried out the first quantitative measurements of aluminium in brain tissue for MS and supported these data with the first imaging of the location of aluminium in donor tissues.

## 2. Materials and Methods

### 2.1. Tissues

Brain tissues were obtained from the Multiple Sclerosis Society Tissue Bank, Imperial College, London following ethical approval (NRES Approval No. 08/MRE09/31). Snap-frozen tissue was obtained from 8 female (39–82 years old) and 6 male (38–66 years old) donors with diagnoses of primary progressive (2), relapsing progressive (2), secondary progressive (9) or relapsing remitting (1) MS [11]. Histology slides were provided from 1 female (SPMS) and 1 male (RRMS) donor. 

### 2.2. Quantitative Measurements

The aluminium content of tissues was measured by an established and fully validated method [12] that herein is described only briefly. Samples of cortex, between 0.6 and 5.0 g in weight, were thawed at room temperature and cut, using a stainless steel blade, into sections approximately 0.3 g in weight. Tissues were dried to a constant dry weight in an incubator at 37 °C. Dry and weighed tissues were digested in a microwave (MARS Xpress CEM Microwave Technology Ltd., Buckingham, UK) in a mixture of 1 mL 15.8 M HNO_3_ (Fisher Analytical Grade, Loughborough, UK) and 1 mL 30% *w/v* H_2_O_2_ (BDH Aristar, Poole, UK). The resultant digests were clear with no fatty residues and, upon cooling, were made up to 5 mL using ultrapure water (cond. < 0.067 μS/cm). Total aluminium was measured in each sample by transversely heated graphite furnace atomic absorption spectrometry (TH GFAAS), Perkin-Elmer, Beaconsfield, UK) using matrix-matched standards and an established analytical programme alongside previously validated quality assurance data including the application of 174 method blanks to account for issues of extraneous contamination [12].

### 2.3. Fluorescence Microscopy

Methods are described in full elsewhere [10] and only briefly summarised herein. Chemicals were purchased from Sigma Aldrich (Poole, UK) unless otherwise stated. The MS Society Brain Bank provided five 5-μm thick serial paraffin-embedded histology sections of brain tissue from frontal, parietal, occipital and temporal lobes and hippocampal tissue from two donors, a female with SPMS and a male with RRMS.

Sections were deparaffinised using Histo-Clear (National Diagnostics, Nottingham, UK), rehydrated through an ethanol gradient and rinsed thoroughly in ultrapure water (cond. < 0.067 μS/cm) using standard techniques, prior to staining. Sections were stained for 45 min in a 50 mM PIPES buffer, pH 7.4 for the assessment of tissue autofluorescence or lumogallion (4-chloro-3-(2,4-dihydroxyphenylazo)-2-hydroxybenzene-1-sulphonic acid, TCI Europe N.V. Belgium), as has been previously optimised for use in cells [13] and human tissues [14]. Sections were subsequently washed in 50 mM PIPES buffer, pH 7.4 and rinsed for 30 s in ultrapure water prior to mounting with Fluoromount™. Fluorescence microscopy was performed using an Olympus BX50 fluorescence microscope equipped with a mercury lamp as the illumination source. Lumogallion fluorescence was acquired using a U-MNIB3 filter cube (λ_ex_: 470–495 nm, DM: 505 nm, λ_em_: 510 nm, longpass, Olympus, Southend-on-Sea, UK) at a final magnification of ×400. Lumogallion stained sections were sequentially scanned in the sequence that lumogallion reactive regions were imaged on adjacent non-stained serial sections. Fluorescence micrographs were obtained using the Cell^D^ software suite (Soft imaging Solutions, SiS, Olympus, GmbH, Münster, Germany) and channels merged by use of Photoshop (Adobe Systems Inc., Palo Alto, CA, USA). The size and morphology of cells and their localisation in specific brain regions were considered when assessing the presence of intracellular aluminium.

### 2.4. Statistical Analyses

Data for aluminium content of tissues were not normally distributed and being strongly skewed were log transformed to allow for the use of parametric tests. The median and interquartile range were calculated for each donor and additionally per donor and lobe. Non-parametric correlation analyses between non-transformed aluminium data and parametric correlations using log-transformed aluminium data and age were performed. Analysis of variance for repeated measurements (stratified for lobes) was used to detect significant differences between lobes, gender and age classes. We considered a p-value smaller than 0.05 to be statistically significant. For both matching and statistical analyses, SPSS Statistics v.22 (IBM Analytics, Armonk, NY, USA) was used. All statistical analyses were done with SPSS.

## 3. Results

### 3.1. Aluminium Content of Brain Tissues

The aluminium content of all tissues ranged from 0.01 (the limit of quantitation) to values in excess of 50 μg/g dry wt. (Supplementary Tables). Where tissues were available for all four main lobes, the aluminium content (Mean ± SD) for whole brains was 6.94 ± 13.75 (MS107), 6.38 ± 23.56 (MS274), 1.55 ± 2.06 (MS356) and 3.32 ± 6.37 μg/g dry wt. (MS401) (Table 1). Detailed statistical analyses (see Section 2.4) of raw and transformed data have not revealed any statistically significant differences between the aluminium content of different lobes in relation to either gender or age of the donor.

Previous measurements of brain aluminium content in our laboratory, using identical analytical methods, have allowed us to define loose categories of brain aluminium content beginning with ≤1.00 μg/g dry wt. as pathologically benign (as opposed to ‘normal’). Approximately 42% of tissues (141/332) fitted this category while about 33% of tissues (108/332) had an aluminium content considered as pathologically concerning (≥2.00 μg/g dry wt.). Almost 80% of these pathologically concerning tissues (83/108) had an aluminium content considered as pathologically significant (≥3.00 μg/g dry wt.). Every individual had at least one tissue with a pathologically significant content of aluminium (Supplementary Tables). The brains of 11 individuals had at least one tissue with an aluminium content ≥ 5.00 μg/g dry wt. while seven of these donors had at least one tissue with an aluminium content ≥ 10.00 μg/g dry wt. (Supplementary Tables).

### 3.2. Aluminium Fluorescence in Brain Tissues

**Figure 1:**
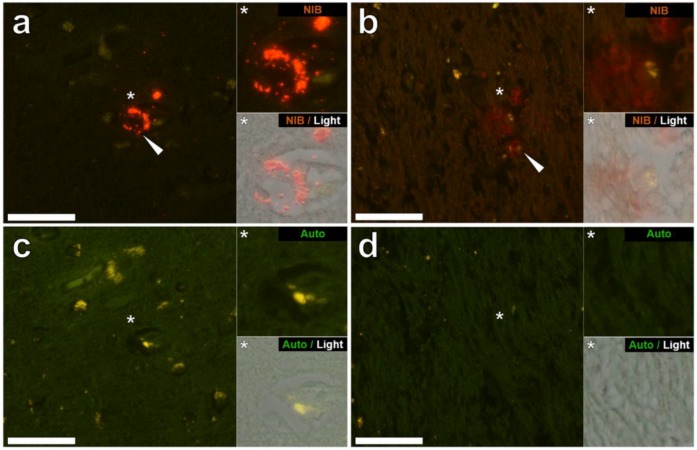
Extracellular aluminium in the frontal lobe and hippocampus of a 56-year-old male donor (MS274), diagnosed with RRMS. (**a**) Intense orange fluorescence (white arrow) indicating punctate deposits of aluminium was observed in the perivascular region of a small blood vessel in the white matter of the frontal lobe, in close proximity to lipofuscin, identified by yellow fluorescence. (**b**) Extracellular deposits of aluminium, identified as diffuse orange-red fluorescence, appear co-deposited with lipofuscin (white arrow) in white matter adjacent to the parahippocampal gyrus. (**c**,**d**) Autofluorescence of serial sections confirms the identity of aluminium in (**a**,**b**) respectively. Upper and lower panels depict magnified inserts of the fluorescence channel and bright field overlay. Magnification ×400, scale bars: 50 μm.

**Figure 2:**
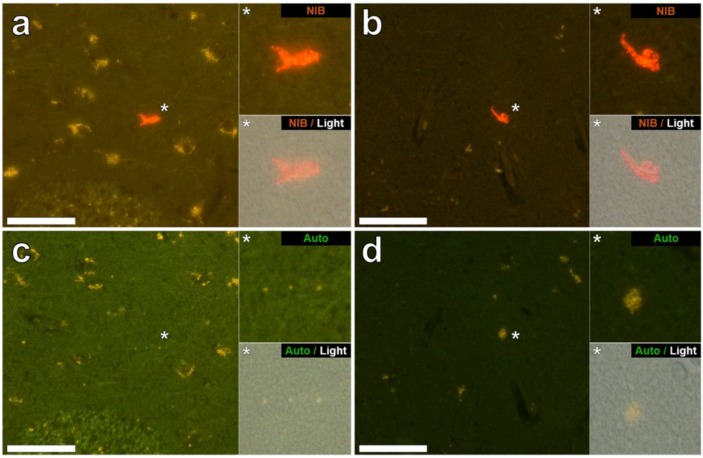
Intracellular aluminium in glia observed in the temporal lobe and hippocampus of a 56-year-old male donor (MS274), diagnosed with RRMS. Bright orange fluorescence was observed intracellularly and co-deposited within the cellular debris of occasional cells morphologically compatible with glial cells in (**a**) the internal capsule and (**b**) the hippocampus (both grey matter). Autofluorescence of serial sections (**c**,**d**) confirms the identity of aluminium in (**a**,**b**) respectively. Upper and lower panels depict magnified inserts of the fluorescence channel and bright field overlay. Magnification ×400, scale bars: 50 μm.

**Figure 3:**
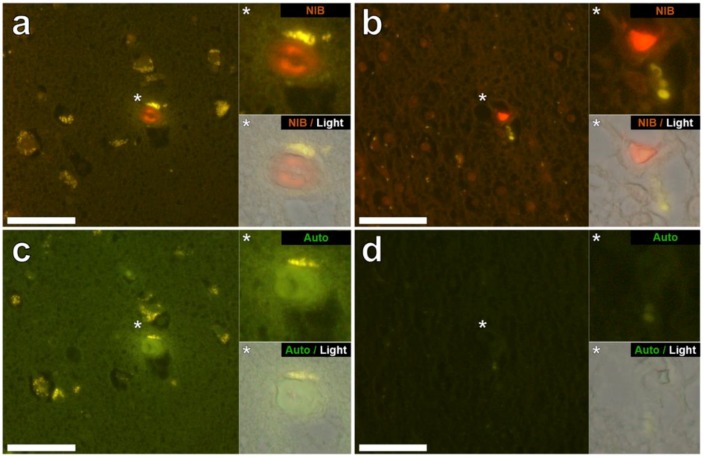
Aluminium in the frontal lobe and hippocampus of a 48-year-old female donor (MS317), diagnosed with SPMS. (**a**) Intense orange aluminium fluorescence was identified in refractile corpora amylacea (or mineralised deposits) in the frontal cortex (grey matter). (**b**) Intracellular aluminium was also observed in occasional glial-like cells in the parahippocampal gyrus (white matter). Autofluorescence of serial sections (**c**,**d**) confirms the identity of aluminium in (**a**,**b**) respectively. Upper and lower panels depict magnified inserts of the fluorescence channel and bright field overlay. Magnification ×400, scale bars: 50 μm.

**Figure 4:**
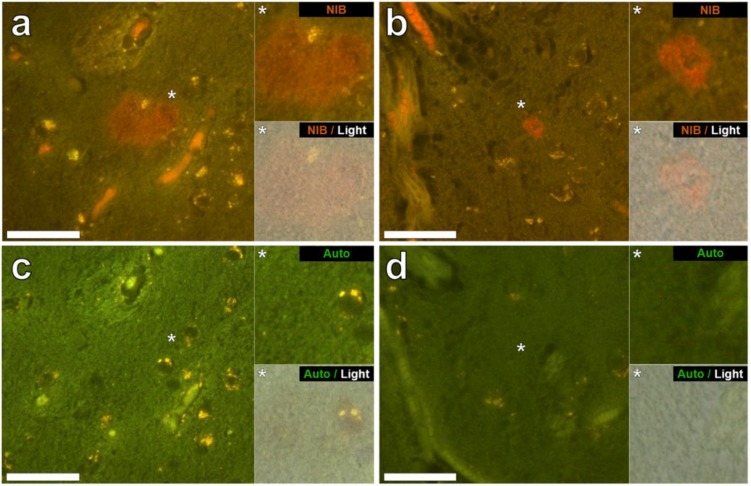
Extracellular aluminium in close-proximity to the temporal lobes of a 56-year-old male donor (MS274) and a 48-year-old female donor (MS317), diagnosed with RRMS and SPMS respectively. (**a**) Diffuse extracellular aluminium fluorescence in the basal ganglia of the male donor spanning ~50 μm in diameter (grey matter). (**b**) Extracellular deposits of aluminium in the medial temporal region of the female donor (grey matter). Autofluorescence of serial sections (**c**,**d**) confirms the identity of aluminium in (**a**,**b**) respectively. Upper and lower panels depict magnified inserts of the fluorescence channel and bright field overlay. Magnification ×400, scale bars: 50 μm.

Aluminium was successfully identified in brain tissue of both donors by aluminium-specific fluorescence microscopy. Significant extracellular accumulations of aluminium were found in association with small blood vessels in the white matter of the frontal lobe of a male donor with RRMS (Figure 1a). Lipofuscin is a granular, ageing-associated, lipid-rich pigment that predominantly accumulates intraneuronally in brain tissue as a product of lysosomal degradation [9]. Herein, lipofuscin deposits were identified across all MS donor tissues via fluorescence microscopy, appearing as an autofluorescent yellow pigment in both lumogallion and non-stained tissue sections (Figure 1a). Autofluorescence imaging of a serial section of tissue confirmed the deposits as aluminium (Figure 1c). Extracellular aluminium was also identified in the parahippocampal gyrus of the same donor (Figure 1b) and was again confirmed by autofluorescence of a serial section (Figure 1d). Intracellular deposits of aluminium, possibly associated with microglia-like cells, were also identified in the temporal lobe (Figure 2a) and hippocampus (Figure 2b) of the same donor with these clear lumogallion-reactive accumulations of aluminium being confirmed by autofluorescence of serial tissue sections (Figure 2c,d). Strong aluminium fluorescence was observed associated with refractile corpora amylacea-like structures in the frontal cortex of a female donor with SPMS (Figure 3a). While strong orange fluorescence confirmed the presence of aluminium, light green autofluorescence from this structure was probably indicative of its refractile nature (Figure 3c). The parahippocampal gyrus of the same donor showed numerous intracellular deposits of aluminium and especially associated with glial, perhaps astrocyte-like cells (Figure 3b). Autofluorescence of a serial section confirmed the presence of aluminium in this tissue (Figure 3d). Diffuse extracellular deposits of aluminium, up to 50 μm across, were identified in the basal ganglia of the male donor with RRMS (Figure 4a) and autofluorescence confirmed its presence as aluminium (Figure 4c). Other unusual extracellular (or possibly intraneuronal) deposits of aluminium were identified in the medial temporal region of the female donor with SPMS (Figure 4b) and the presence of aluminium was confirmed by autofluorescence (Figure 4d). Many areas of brain tissue from both donors showed no positive staining for aluminium (Supplementary Figure S1).

## 4. Discussion

We have made the first measurements of aluminium in brain tissue from donors with diagnoses of MS. Each of the 14 donors had at least one tissue with a pathologically significant (≥3.00 μg/g dry wt.) concentration of aluminium (see Supplementary Tables). While there were clear differences in the aluminium content of different lobes in the same individual, for example, male donor MS107 or female donor MS401 (Table 1), overall there were no statistically significant differences relating to the distribution and accumulation of aluminium in MS brain tissue. Statistical analyses were hampered by tissues being unavailable for some lobes in some individuals and the varied wet weights of supplied tissues. The ages of donors were relatively young, 11/14 were below the age of 60 and the presence of high concentrations of aluminium in younger individuals was an unexpected finding (See Supplementary Tables). For example, concentrations of 40.56 and 63.13 μg/g dry wt. in a 38-year-old male donor (MS107) were remarkable. These measurements add to our burgeoning database of aluminium in human brain tissue [9,10,12,14,15,16] and reinforce the conclusion of House et al. [12] that there is more aluminium in human brain tissue than perhaps previously thought [17]. What is lacking are data representing true control tissues, the aluminium content of brain tissue prior to any sign of neurodegeneration, and we will address this gap in knowledge in the near future. However, the data herein do suggest above normal content of brain aluminium in MS and as such may partially explain the high levels of urinary aluminium excretion in MS [5,6].

Aluminium-specific fluorescence [14] located aluminium in the brain tissue of a male (MS274) and female (MS317) donor (Figure 1, Figure 2, Figure 3 and Figure 4). In both of these donors the lobes, specifically frontal and temporal, where aluminium was shown to be high by TH GFAAS (Table 1), also showed many more deposits of aluminium by fluorescence. Aluminium was found in both extracellular and intracellular locations and in both grey and white matter. It was difficult to associate the aluminium with any known pathologies specific to MS though aluminium was found in diffuse, perhaps plaque-like structures (Figure 4), glial-like cells, as well as in corpora amylacea (Figure 3). The latter was of particular interest since corpora amylacea are possible tombstones of neurodegeneration and in MS could be remnants of both neuronal and non-neuronal cell death [18,19]. Aluminium was located throughout the hippocampus of both donors (Figure 1 and Figure 3) tissues, which were not made available for quantitative analyses. It is important to stress that large areas of all tissues studied for these donors did not show any positive aluminium-specific fluorescence (Supplementary Figure S1).

## 5. Conclusions

We have investigated the aluminium content of brain tissue in MS and shown the aluminium content to be elevated and associated with the condition including neurodegeneration. Aluminium may contribute towards the aetiology of MS and recent research demonstrating the efficacy of silicon-rich mineral waters in increasing the urinary excretion of aluminium [6] may represent a therapeutic strategy for individuals with MS.

## Figures and Tables

**Table 1:** Brain aluminium content (μg/g dry wt. mean (SD) (n)) for each lobe of each MS donor. NA—tissue unavailable; PPMS—primary progressive MS; RPMS—relapsing progressive MS; SPMS—secondary progressive MS; RRMS—relapsing remitting MS.

Donor Id.	Gender	Age	MS	Frontal	Temporal	Occipital	Parietal
MS307	Male	55	PPMS	1.75(0.91) (5)	0.46(0.60) (13)	NA	1.76(1.28) (6)
MS107	Male	38	RPMS	3.41(3.54) (5)	0.59(0.64) (3)	0.58(0.04) (2)	9.84(16.70) (17)
MS245	Male	64	SPMS	2.86(0.80) (4)	NA	NA	NA
MS274	Male	56	RRMS	29.14(57.92) (5)	3.53(2.55) (13)	0.50(0.57) (10)	0.36(0.20) (3)
MS304	Male	52	SPMS	1.82(1.40) (5)	NA	3.98(9.84) (28)	NA
MS313	Male	66	PPMS	0.47(0.63) (5)	NA	3.64(4.41) (20)	1.28(3.22) (20)
MS330	Female	59	SPMS	2.02(1.64) (5)	NA	NA	NA
MS317	Female	48	SPMS	5.44(5.73) (5)	NA	NA	NA
MS356	Female	45	SPMS	1.84(2.85) (5)	1.81(1.78) (5)	1.40(1.86) (16)	1.51(2.44) (8)
MS401	Female	82	SPMS	0.65(0.65) (4)	1.55(1.96) (16)	5.66(9.27) (20)	2.36(1.65) (8)
MS180	Female	44	SPMS	3.34(3.71) (5)	NA	NA	NA
MS114	Female	52	SPMS	1.68(1.25) (5)	3.45(4.38) (17)	NA	0.59(0.55) (6)
MS203	Female	53	SPMS	2.39(1.34) (5)	0.81(0.41) (7)	NA	3.31(4.00) (2)
MS234	Female	39	RPMS	6.35(8.27) (5)	NA	0.74(0.50) (5)	2.79(2.83) (19)

## Supplementary Materials

The following are available online at http://www.mdpi.com/1660-4601/15/8/1777/s1, Figure S1: Representative negative lumogallion staining of white and grey matter of the occipital lobe of a 56-year-old male donor (MS274), diagnosed with RRMS. T, Tables S1: MS307 (Male, 55 PPMS), Table S2: MS107 (Male, 38 RPMS), Table S3: MS245 (Male, 64 SPMS), Table S4: MS274 (Male, 56 RRMS), Table S5: MS330 (Female, 59 SPMS), Table S6: MS317 (Female, 48 SPMS), Table S7: MS304 (Male, 52 SPMS), Table S8: MS356 (Female, 45 SPMS), Table S9: MS401 (Female, 82 SPMS), Table S10: MS180 (Female, 44 SPMS), Table S11: MS313 (Male, 66 PPMS), Table S12: MS114 (Female, 52 SPMS), Table S13: MS203 (Female, 53 SPMS), Table S14: MS234 (Female 39, RPMS).
