# Supplementary material for: Aluminium in Brain Tissue in Multiple Sclerosis
Source: Int J Environ Res Public Health. 2018 Aug 18;15(8):1777. doi: 10.3390/ijerph15081777 (PMC6121957; doi:10.3390/ijerph15081777)
Supplement: Supplementary file 1 [file ijerph-15-01777-s001.pdf]

International Journal of
Environmental Research and Public Health

MDPI

Supplementary material

Aluminium in Brain Tissue in Multiple Sclerosis

Matthew Mold [1], Agata Chmielecka [2], Maria Raquel Ramirez Rodriguez [1], Femia Thom [2], Caroline Linhart [3], Andrew King [4] and Christopher Exley [1,*]

[1] The Birchall Centre, Lennard-Jones Laboratories, Keele University, UK; m.j.mold@keele.ac.uk (M.M.); raquel.ramirez3@hotmail.com (M.R.R.R.)
[2] Life Sciences, The Huxley Building, Keele University, UK; aggychmi@gmail.com (A.C.); femiathom@hotmail.com (F.T.)
[3] Department of Medical Statistics, Informatics and Health Economics, Medical University of Innsbruck, Austria; Linhart.Caroline@i-med.ac.at
[4] Department of Clinical Neuropathology, Kings College Hospital, London, UK; andrewking@nhs.net
* Correspondence: c.exley@keele.ac.uk

Received: 25 July 2018; Accepted: 15 August 2018; Published: date

Supplementary Figure 1. Brain tissue found negative for the presence of aluminium when stained with lumogallion.

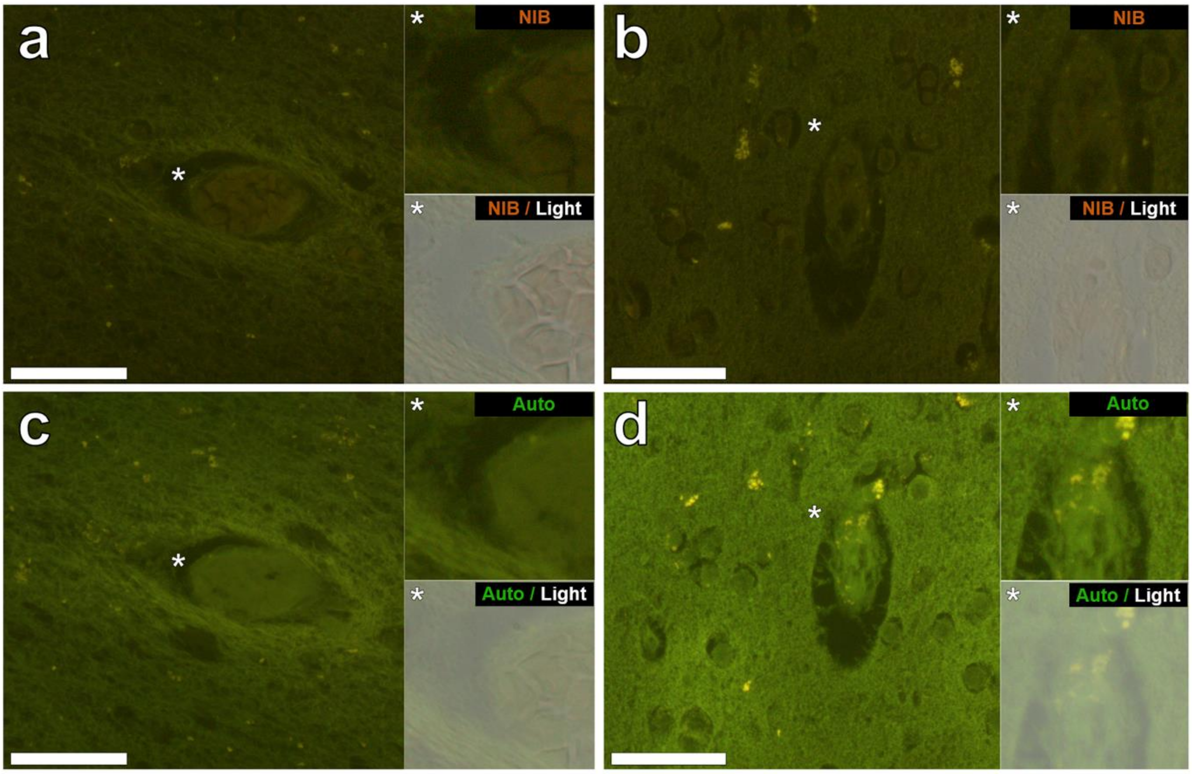

Supplementary Figure 1. Representative negative lumogallion staining of white and grey matter of the occipital lobe of a 56-year-old male donor (MS274), diagnosed with RRMS. Lumogallion staining of white (a) and grey (b) matter of the hippocampus produced dull brown fluorescence associated with blood vessels and distinguishable from yellow punctate fluorescence of lipofuscin deposits. Green autofluorescence was observed in adjacent non-stained serial sections (c & d). Upper and lower panels depict magnified inserts of the fluorescence channel and the bright field overlay. Magnification X 400, scale bars: 50µm.

Supplementary Tables 1 – 14. Tables include aluminium data (μg/g dry wt.) for all measured tissues for each lobe for each MS donor.

Table S1. MS307 (Male, 55 PPMS)

Frontal	Temporal	Occipital	Parietal
2.00	1.08	NA	0.52
3.25	0.01		2.36
1.20	0.69		0.49
1.20	1.34		3.67
1.12	0.67		2.47
	0.01		1.03
	0.42		
	0.01		
	0.01		
	0.01		
	0.01		
	0.01		
	1.76		
1.75 (0.91)	0.46 (0.60)		1.76 (1.28)

Table S2. MS107 (Male, 38 RPMS)

Frontal	Temporal	Occipital	Parietal
0.18	0.01	0.55	3.29
7.92	1.28	0.61	2.66
6.51	0.49		6.04
1.60			2.57
0.85			40.56
			12.80
			7.97
			0.15
			6.85
			7.77
			63.13
			0.01
			0.01
			8.26
			0.56
			2.75
			1.81
3.41 (3.54)	0.59 (0.64)	0.58 (0.04)	9.84 (16.70)

Table S3. MS245 (Male, 64 SPMS)

Frontal	Temporal	Occipital	Parietal
2.75			
2.73			
3.93			
2.01			
2.86 (0.80)			

Table S4. MS274 (Male, 56 RRMS)

Frontal	Temporal	Occipital	Parietal
132.64	6.52	1.43	0.52
7.48	4.13	1.67	0.42
0.47	1.69	0.37	0.13
3.61	0.34	0.14	
1.51	3.03	0.01	
	2.36	0.39	
	1.73	0.18	
	3.47	0.12	
	1.91	0.35	
	1.43	0.34	
	3.08		
	7.37		
	8.84		
29.14 (57.92)	3.53 (2.55)	0.50 (0.57)	0.36 (0.20)

Table S5. MS330 (Female, 59 SPMS)

Frontal	Temporal	Occipital	Parietal
0.55			
4.43			
1.17			
3.00			
0.94			
2.02 (1.64)			

Table S6. MS317 (Female, 48 SPMS)

Frontal	Temporal	Occipital	Parietal
1.91			
13.73			
1.32			
9.18			
1.05			
5.44 (5.73)			

Table S7. MS304 (Male, 52 SPMS)

Frontal	Temporal	Occipital	Parietal
2.90	NA	3.47	NA
3.65		1.88	
1.38		0.01	
0.68		1.83	
0.47		3.15	
		1.19	
		1.19	
		8.09	
		1.91	
		3.85	
		1.10	
		0.21	
		1.84	
		52.98	

	7.63
	1.14
	1.00
	2.89
	7.13
	0.48
	2.30
	1.67
	0.42
	1.02
	1.11
	1.16
	0.22
	0.61
1.82 (1.40)	3.98 (9.84)

Table S8. MS356 (Female, 45 SPMS)

Frontal	Temporal	Occipital	Parietal
6.84	1.75	0.18	0.01
0.29	1.46	0.32	1.17
1.46	0.19	0.49	0.01
0.01	4.81	1.25	0.01
0.61	0.84	0.05	0.01
		0.95	1.72
		1.79	1.92
		1.09	7.21
		1.73	
		0.38	
		2.53	
		0.23	
		7.07	
		0.28	
		0.01	
		4.00	
1.84 (2.85)	1.81 (1.78)	1.40 (1.86)	1.51 (2.44)

Table S9. MS401 (Female, 82 SPMS)

Frontal	Temporal	Occipital	Parietal
0.47	1.21	0.40	4.87
1.56	0.18	0.62	3.69
0.01	1.95	0.65	1.96
0.55	1.11	1.58	1.30
	0.52	0.59	0.71
	0.60	1.33	4.25
	0.50	1.87	1.21
	1.19	1.61	0.91
	0.36	7.12	
	8.05	29.32	
	1.13	0.88	
	4.10	0.70	
	0.60	2.44	
	1.54	0.20	
	0.96	27.87	
	0.83	2.33	

		0.01	
		0.36	
		14.90	
		18.42	
0.65 (0.65)	1.55 (1.96)	5.66 (9.27)	2.36 (1.65)

Table S10. MS180 (Female, 44 SPMS)

Frontal	Temporal	Occipital	Parietal
0.59	NA	NA	NA
1.17			
1.72			
9.68			
3.52			
3.34 (3.71)			

Table S11. MS313 (Male, 66 PPMS)

Frontal	Temporal	Occipital	Parietal
0.34	NA	8.70	0.99
0.01		3.11	0.01
0.01		0.32	1.01
1.53		10.30	0.01
0.45		3.47	0.01
		1.85	0.01
		5.15	0.01
		5.39	0.01
		18.13	0.01
		3.68	14.24
		1.71	0.95
		0.56	1.02
		1.22	0.01
		1.59	0.01
		3.50	4.30
		0.12	1.83
		3.16	0.01
		0.68	0.29
		0.01	0.01
		0.23	0.78
0.47 (0.63)		3.64 (4.41)	1.28 (3.22)

Table S12. MS114 (Female, 52 SPMS)

Frontal	Temporal	Occipital	Parietal
0.53	1.15	NA	0.26
1.40	0.36		0.28
3.70	1.78		0.41
1.95	1.92		0.10
0.82	17.61		0.92
	1.31		1.55
	0.72		
	0.01		
	0.01		
	8.24		
	5.94		

	0.51		
	4.17		
	6.67		
	2.17		
	3.48		
	2.54		
1.68 (1.25)	3.45 (4.38)		0.59 (0.55)

Table S13. MS203 (Female, 53 SPMS)

Frontal	Temporal	Occipital	Parietal
2.50	1.00	NA	6.13
1.30	0.17		0.49
1.39	1.41		
4.61	0.62		
2.15	1.03		
	0.47		
	0.94		
2.39 (1.34)	0.81 (0.41)		3.31 (4.00)

Table S14. MS234 (Female 39, RPMS)

Frontal	Temporal	Occipital	Parietal
2.67	NA	0.65	3.21
2.11		1.37	0.90
21.12		1.13	1.14
3.09		0.24	9.69
2.74		0.31	0.84
			7.52
			5.02
			1.14
			2.49
			0.01
			0.01
			3.70
			0.01
			2.12
			0.01
			0.34
			4.09
			4.25
			6.57
6.35 (8.27)		0.74 (0.50)	2.79 (2.83)
